# Supplementary material for: Pulmonary and Systemic Immune Profiles Following Lung Volume Reduction Surgery and Allogeneic Mesenchymal Stromal Cell Treatment in Emphysema
Source: Cells. 2024 Sep 30;13(19):1636. doi: 10.3390/cells13191636 (PMC11476308; doi:10.3390/cells13191636)
Supplement: Supplementary file 1 [file cells-13-01636-s001.zip › cells-3178937-supplementary.pdf]

Supplemental Material and data

Pulmonary and systemic immune profiles upon lung volume reduction surgery and allogeneic mesenchymal stromal cell treatment in emphysema

Li Jia[1,2], Na Li[1,3], Vincent van Unen[1], Jaap-Jan Zwaginga[4], Jerry Braun[5], Pieter S. Hiemstra[2], Frits Koning[1], P. Padmini S.J. Khedoe[2], Jan Stolk[2]

Supplemental Material and Methods

Patient selection

This study was registered at clintrials.gov under NCT04918706 and was approved by the Central Committee on Research Involving Human Subjects (CCMO) of The Netherlands; all study participants provided written informed consent. COPD patients with emphysema were included in the study with the following inclusion criteria: the gradient of emphysema was equally distributed between left and right lung; a forced expiratory volume (FEV_1) between 20-45 of % predicted; a diffusing capacity of the lungs for carbon monoxide/ VA (Kco) between 24-67 of % predicted and a residual volume/ lung capacity or total lung capacity (RV/ TLC) ≥ 50%. Included participants had stopped smoking at least one year prior to the first screening visit and were in stable clinical condition to be eligible for lung volume reduction surgery (LVRS). All included emphysema patients had the MM genotype of SERPINA1, and therefore alpha1-antitrypsin deficiency (AATD) was excluded. The first randomized patient in the study resumed cigarette smoking about 2 weeks after L1 and was excluded from further participation and did not receive MSC/ placebo treatment.

The exclusion criteria were: significant cardiac failure; active smoking, or <6 months of smoking cessation; or failure to complete a pulmonary rehabilitation program before study randomization; or women of child bearing potential; or any other condition of the patient that the clinical investigator deemed harmful for study participation for NCT04918706.

Next, lung densitometry of images of chest Computed Tomography (CT) without contrast medium was performed as previously described to assess if emphysema was of similar severity in both lungs, in order to perform LVRS in patients which had emphysema in the upper lobe with similar low tissue density between the right and left lung lobe.[1] Finally, myocardial perfusion scintigraphy with dobutamine-induced pharmacological stress was performed using Tc-99m Tetrofosmin i.v. SPECT-

acquisitions over 360° that were used for cardiac reconstruction to measure left ventricular ejection fraction and to identify myocardial ischaemia in order to exclude patients with cardiac failure[2].

The cell product for this study was produced under GMP-conditions and is a LUMC-initiated thoroughly characterized cell suspension of allogeneic MSC isolated from bone marrow of healthy donors (BM-MSC)[3]. BM-MSC were expanded *ex vivo* and were cryopreserved before administration. Emphysema patients received placebo, 100 ml 0.9% NaCl/ 5% DMSO (n=5) or 2 x 10^6 cells/kg body weight BM-MSC (n=9) in 100 ml 0.9% NaCl/ 5% DMSO, with a lower limit of 1.5 x 10^6 cells/kg.

Randomization was performed by software operated by the responsible QP from the LUMC Pharmacy, section of cell therapy. All investigators and patients were blinded to treatment during the study and during follow-analyses and measurement.

Trial power analysis: to obtain 80% power and a two-sided significance level of 0.05, we calculated that 10 patients were required for treatment with BM-MSC based on CD31 IHC expression data. (CD31 expression in alveolar septa measured in tissue from historic controls was 0.02835 ± 0.01648 /µm (mean ± SD) and of BM-MSC-treated patients from the pilot study was 0.06607 ± 0.02342 /µm).

Tissue and blood processing

Resected lung tissue was washed with HBSS (Thermofisher, UK) 6-8 times to remove excess blood. Then the tissue was cut into small pieces for enzymatic digestion in 30 mL IMDM (Lonza, Basel, Switzerland) supplemented with 10% FCS (BODINCO BV, Netherlands), 300 U/mL Collagenase IV (Worthington Biochemical corporation, USA), 200 µg/mL DNase I (Roche Diagnostics, Basel, Switzerland), 1 µl/mL gentamycin and 2 µl/mL fungizone at 37°C for 2 hours (h). After digestion, the lung cell suspension was filtered through a 70 µm nylon cell strainer (FALCON, USA) and thereafter washed with 0.5% FCS/PBS buffer. Lung mono-nuclear cells were isolated using Ficoll-Paque™ density-gradient centrifugation from digested single lung cells, then

counted using eosin and cryopreserved at a concentration of $3x10^6$ cells/mL in liquid nitrogen until analysis.

Peripheral blood mononuclear cells (PBMCs) were isolated from 8 ml of freshly drawn heparin anticoagulated arterial blood using Ficoll-Paque™ density-gradient centrifugation, and cryopreserved at a concentration of $3x10^6$ cells/ mL in liquid nitrogen until analysis.

Mass cytometry antibody staining and data analysis

Procedures for mass cytometry were performed as described before.[4] In short, lung cells and PBMCs were thawed in IMDM + 50% FCS. Then the cells were incubated with 10 mL IMDM + 200 μg/ml DNase I for 30 minutes at room temperature to remove clumps of dead cells. After washing and centrifugation, the cells were incubated with intercalator-Rh (Fluidigm, USA) for 15 min at RT to stain the dead cells, and then blocked with Fc block buffer to decrease the unspecific binding and lastly stained with mass cytometry surface antibodies for lymphoid and myeloid cells as described before[4]. For intracellular antibody staining, following surface antibody staining, cells were permeabilized with Permeabilization Perm buffer, and thereafter incubated with an intracellular antibody mixture for 30 min at RT. Next, cells were washed and stained for DNA by incubating with intercalator-IR overnight at 4°C. The next day, cell concentration of all samples were adjusted to 0.6 $x10^6$ cells/mL in distilled water for CyTOF measuring. 10% EQ beads were added into the cell samples for data normalization and measured in the Helios time-of-flight mass cytometry (Fluidigm, USA). The experiments were measured in 9 batches, and a reference PBMC sample was included in each batch as staining control and for normalization.

References

1. Mascalchi M, Camiciottoli G, Diciotti S. Lung densitometry: why, how and when. *J Thorac Dis* 2017;9(9):3319-45. doi: 10.21037/jtd.2017.08.17

2. Elhendy A, Sozzi FB, Valkema R, et al. Dobutamine technetium-99m tetrofosmin SPECT imaging for the diagnosis of coronary artery disease in patients with limited exercise capacity. *J Nucl Cardiol* 2000;7(6):649-54. doi: 10.1067/mnc.2000.109660
3. Reinders ME, Roemeling-van Rhijn M, Khairoun M, et al. Bone marrow-derived mesenchymal stromal cells from patients with end-stage renal disease are suitable for autologous therapy. *Cytotherapy* 2013;15(6):663-72. doi: 10.1016/j.jcyt.2013.01.010 [published Online First: 20130216]
4. Jia L, Li N, Abdelaal TRM, et al. High-Dimensional Mass Cytometry Reveals Emphysema-associated Changes in the Pulmonary Immune System. *Am J Respir Crit Care Med* 2024 doi: 10.1164/rccm.202303-0442OC [published Online First: 20240327]

Supplementary Sfigures

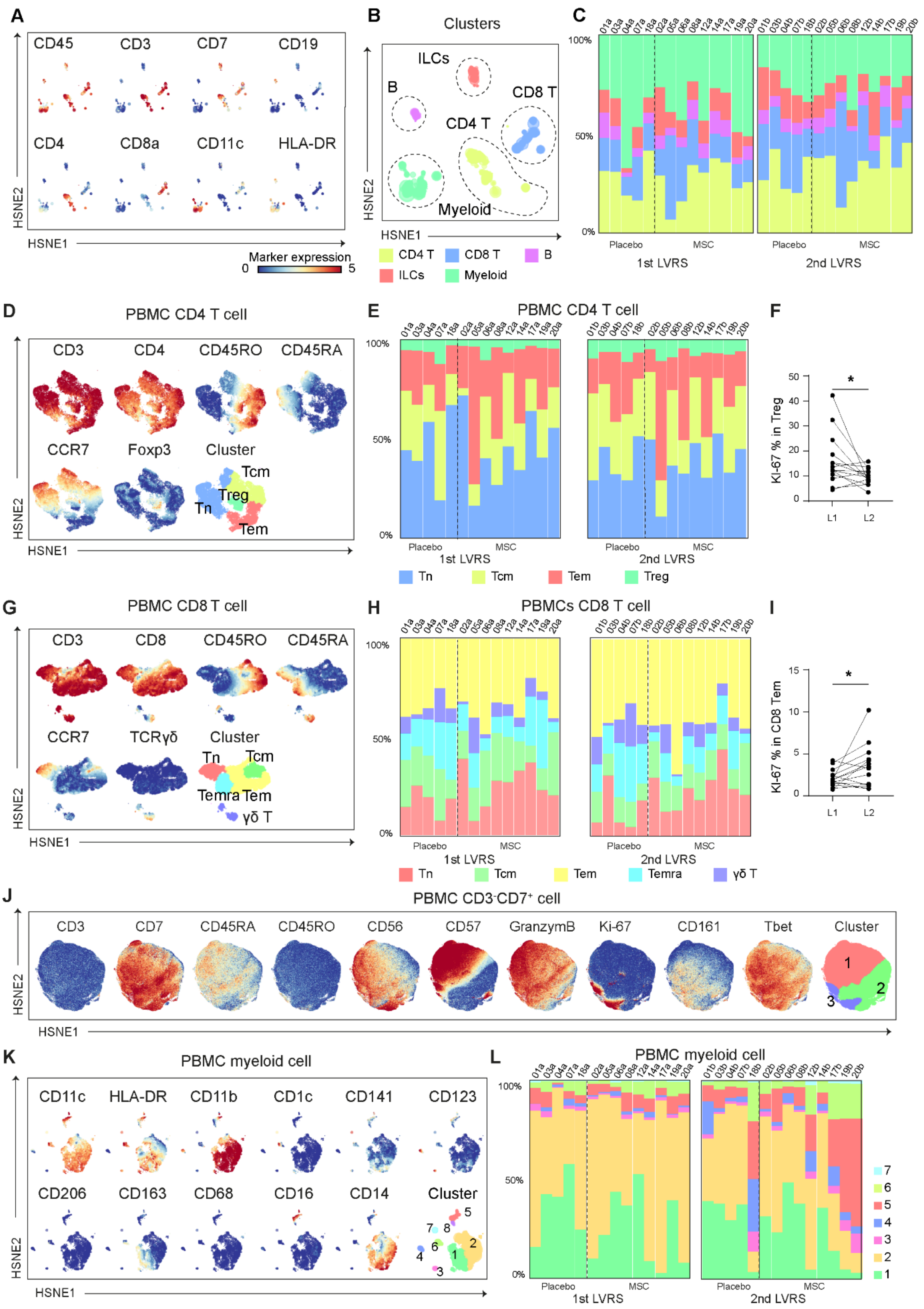

Figure S1. Mass cytometry analysis of circulating immune cells collected from emphysema patients that underwent LVRS and BM-MSC/ placebo treatment

(A) A collective HSNE analysis was performed on $CD45^+$ cells (total 9×10^6 cells) derived from PBMC across L1 and L2, and based on the expression of major immune markers, we identified (B) CD4 T cells, CD8 T cells, B cells, $CD3^-CD7^+$ innate lymphoid cells (ILCs) and myeloid cells. (C) The immune composition in blood from individual patients who underwent L1 and L2 is represented by vertical bars. The length of the coloured segment represents the proportion of cells as percentage of $CD45^+$ PBMCs in each sample. Colours represent different populations within the $CD45^+$ cells. (D) A collective HSNE analysis was performed on the CD4 T cell population derived from PBMCs across L1 and L2. Tn, Tcm, Tem, Temra, Treg subpopulations were visualized in a HSNE plot. (E) The composition of the CD4 T cell subpopulations in patients who underwent L1 and L2 is represented by vertical bars. The length of coloured segment represents the proportion of cells as percentage of CD4 T cell population in each sample. Colours represent different populations within the CD4 T cell population. (F) Quantification of the proportion of Ki-67^+ proliferating Treg cells derived from PBMCs. (G) A collective HSNE analysis was performed on the CD8 T cell population derived from PBMCs across L1 and L2. Tn, Tcm, Tem, Temra, $\gamma\delta$ T subpopulations were visualized in a HSNE plot. (H) The composition of the CD8 T cell subpopulations in patients who underwent L1 and L2 is represented by vertical bars. The length of coloured segment represents the proportion of cells as percentage of CD8 T cell population in each sample. Different colours represent different populations within CD8 T cell population. (I) Quantification the proportion of proliferating CD8 Tem cells in all CD8 Tem cells from PBMCs. (J) A collective HSNE analysis was performed on $CD3^-CD7^+$ innate lymphoid cells derived from PBMCs across L1 and L2. Phenotypical subpopulations were visualized in a HSNE plot. (K) A collective HSNE analysis was performed on the myeloid cell population derived from PBMCs across L1 and L2. Phenotypical subpopulations were visualized in a HSNE plot. (L) The composition of the myeloid cell subpopulations in patients who underwent L1 and L2 is represented

by vertical bars. The length of coloured segment represents the proportion of cells as percentage of CD8 T cell population in each sample. Different colours represent different populations within myeloid cell population.

*$P < 0.05$, **$P < 0.01$, ***$P < 0.001$ by Wilcoxon matched-pairs signed rank test.

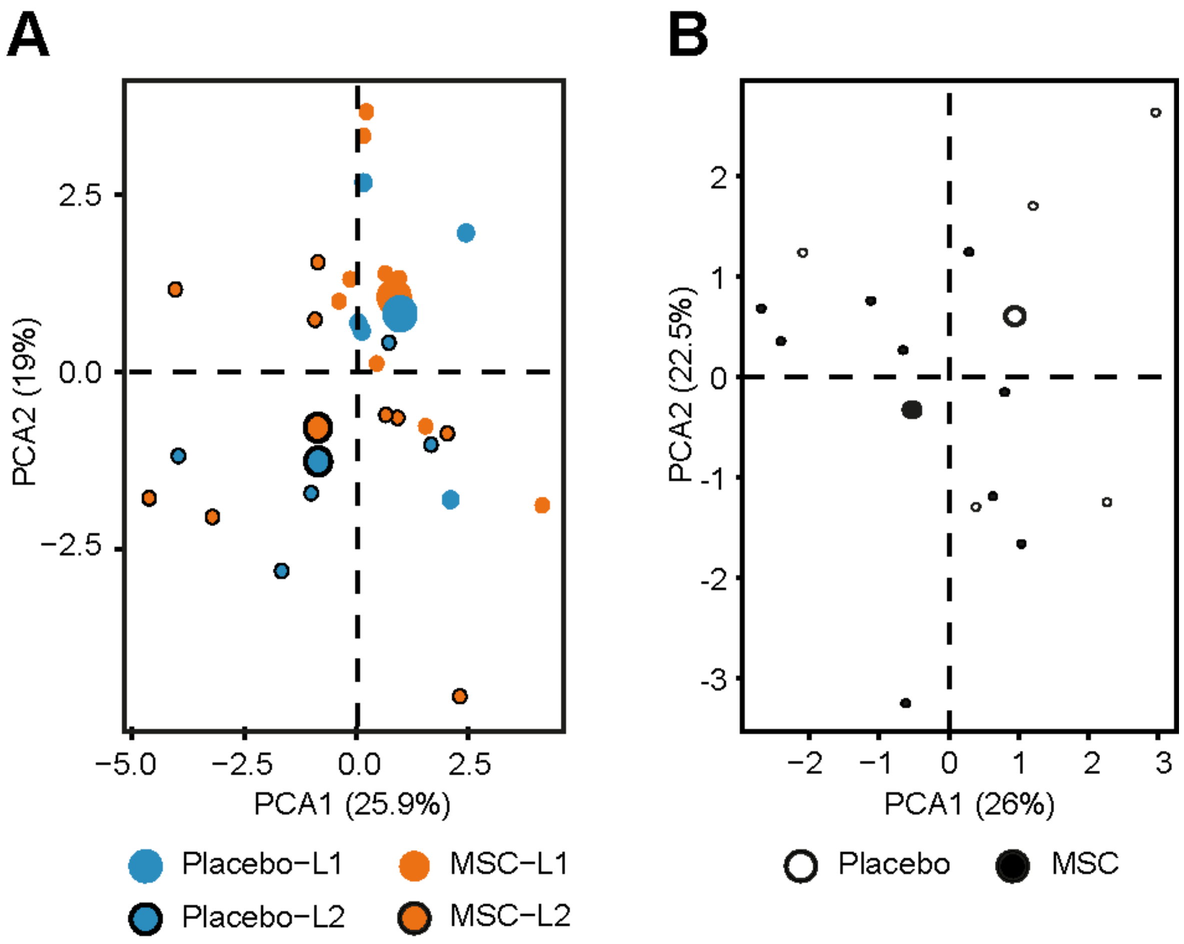

Figure S2. Principal component analysis (PCA) on circulating immune subpopulations

(A) PCA was performed on all the circulating lymphoid and myeloid immune cells in PBMC samples collected from emphysema patients at L1 and L2 and treated with BM-MSC (orange) or placebo (blue). (B) PCA was performed on the differences of significantly altered circulating immune subpopulations (shown in Figure 3B-D) in blood samples collected at L2 compared to L1 to determine the effect of BM-MSC (white dots) or placebo (black dots) treatment.

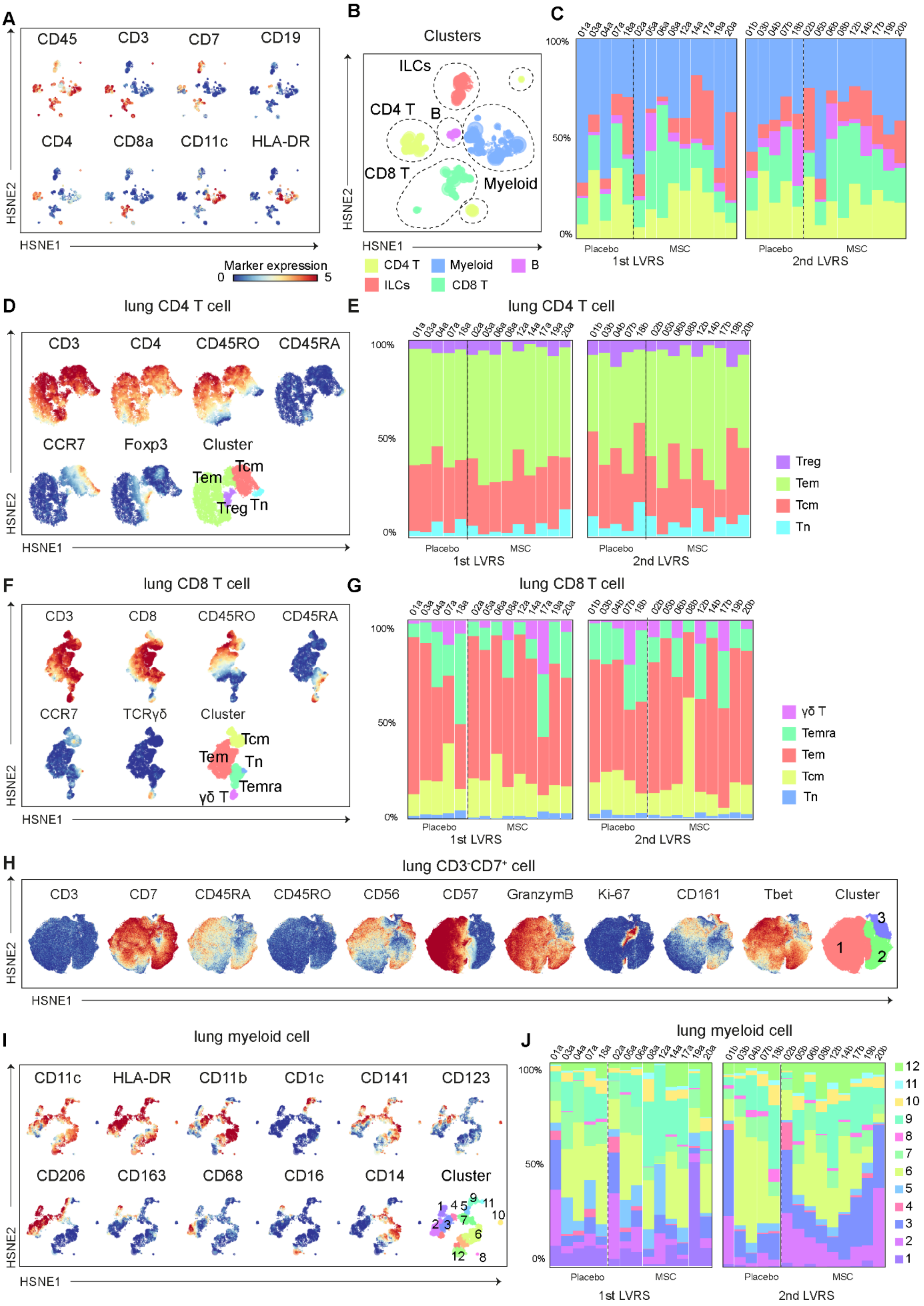
A
CD45
CD3
CD7
CD19
CD4
CD8a
CD11c
HLA-DR
HSNE2
HSNE1
Marker expression
0
5
B
Clusters
ILCs
CD4 T
B
CD8 T
Myeloid
HSNE2
HSNE1
CD4 T
Myeloid
B
ILCs
CD8 T
C
100%
50%
0%
Placebo
MSC
1st LVRS
Placebo
MSC
2nd LVRS
D
lung CD4 T cell
CD3
CD4
CD45RO
CD45RA
CCR7
Foxp3
Cluster
Tem
Tcm
Treg
Tn
HSNE2
HSNE1
E
lung CD4 T cell
100%
50%
0%
Placebo
MSC
1st LVRS
Placebo
MSC
2nd LVRS
Treg
Tem
Tcm
Tn
F
lung CD8 T cell
CD3
CD8
CD45RO
CD45RA
CCR7
TCRγδ
Cluster
Tcm
Tem
Tn
Temra
γδ T
HSNE2
HSNE1
G
lung CD8 T cell
100%
50%
0%
Placebo
MSC
1st LVRS
Placebo
MSC
2nd LVRS
γδ T
Temra
Tem
Tcm
Tn
H
lung CD3⁻CD7⁺ cell
CD3
CD7
CD45RA
CD45RO
CD56
CD57
GranzymB
Ki-67
CD161
Tbet
Cluster
1
2
3
HSNE2
HSNE1
I
lung myeloid cell
CD11c
HLA-DR
CD11b
CD1c
CD141
CD123
CD206
CD163
CD68
CD16
CD14
Cluster
HSNE2
HSNE1
J
lung myeloid cell
100%
50%
0%
Placebo
MSC
1st LVRS
Placebo
MSC
2nd LVRS
12
11
10
9
8
7
6
5
4
3
2
1

Figure S3. Mass cytometry analysis of pulmonary immune cells collected from emphysema patients.

(A) A collective HSNE analysis was performed on $CD45^+$ cells (total 6.2 $\times 10^6$ cells) derived from lung tissue of emphysema patients collected at L1 and L2, and based on the expression of major immune markers, we identified (B) CD4 T cells, CD8 T cells , B cells, $CD3^-CD7^+$ innate lymphoid cells (ILCs) and myeloid cells. (C) The pulmonary immune composition from patients who underwent L1 and L2 is represented by vertical bars. The length of the coloured segment represents the proportion of cells as percentage of $CD45^+$ lung cells in each sample. Different colours represent different populations within the $CD45^+$ cells. (D) A collective HSNE analysis was performed on the CD4 T cell population derived from lungs across L1 and L2. Tn, Tcm, Tem, Temra, Treg subpopulations were visualized in a HSNE plot. (E) The composition of the CD4 T cell subpopulations in patients who underwent L1 and L2 is represented by vertical bars. The length of coloured segment represents the proportion of cells as percentage of CD4 T cell population in each sample. Different colours represent different populations within the CD4 T cell population. (F) A collective HSNE analysis was performed on the CD8 T cell population derived from lungs across L1 and L2. Tn, Tcm, Tem, Temra, $\gamma\delta$ T subpopulations were visualized in a HSNE plot. (G) The composition of the CD8 T cell subpopulations in patients who underwent L1 and L2 is represented by vertical bars. The length of coloured segment represents the proportion of cells as percentage of CD8 T cell population in each sample. Different colours represent different populations within the CD8 T cell population. (H) A collective HSNE analysis was performed on $CD3^-CD7^+$ innate lymphoid cells derived from lungs across L1 and L2. Phenotypical subpopulations were visualized in a HSNE plot. (I) A collective HSNE analysis was performed on the myeloid cell population derived from lungs across L1 and L2. Phenotypical subpopulations were visualized in a HSNE plot. (J) The composition of the myeloid cell subpopulations in patients who underwent L1 and L2 is represented by vertical bars. The length of coloured segment represents the proportion of cells as percentage of CD8 T cell population in each

sample. Different colours represent different populations within myeloid cell population.

*$P < 0.05$, **$P < 0.01$, ***$P < 0.001$ by Wilcoxon matched-pairs signed rank test.

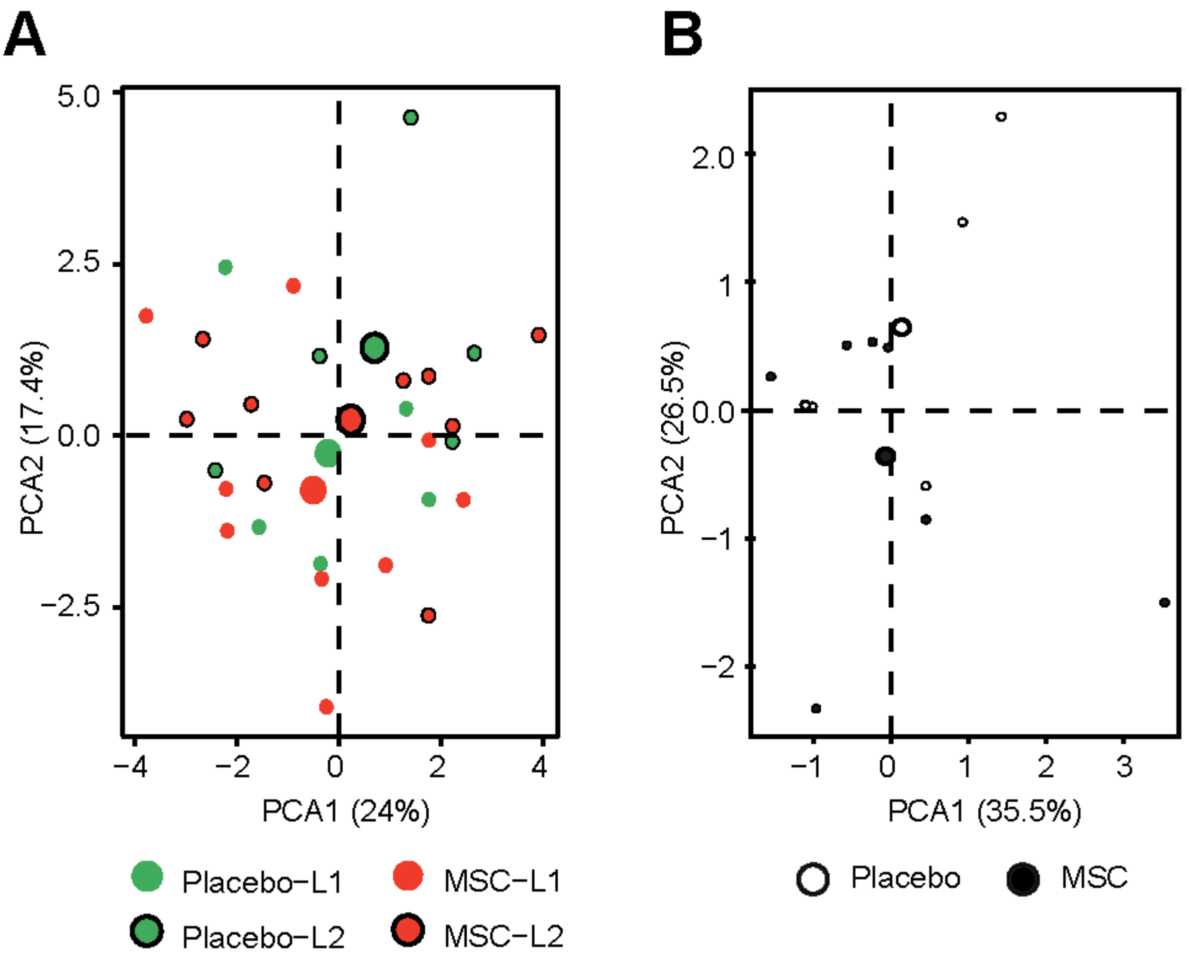

Figure S4. Principal component analysis (PCA) on pulmonary immune subpopulations

(A) PCA was performed on all pulmonary lymphoid and myeloid immune cells derived from lung tissue samples, collected at L1 and L2, of emphysema patients, treated with BM-MSC (red) or placebo (green). (B) PCA was performed on the differences of significantly altered pulmonary immune subpopulations (shown in Figure 4B-D) at lung tissue samples collected at L2 compared to L1, to determine the effect of BM-MSC (white dots) or placebo (black dots) treatment.

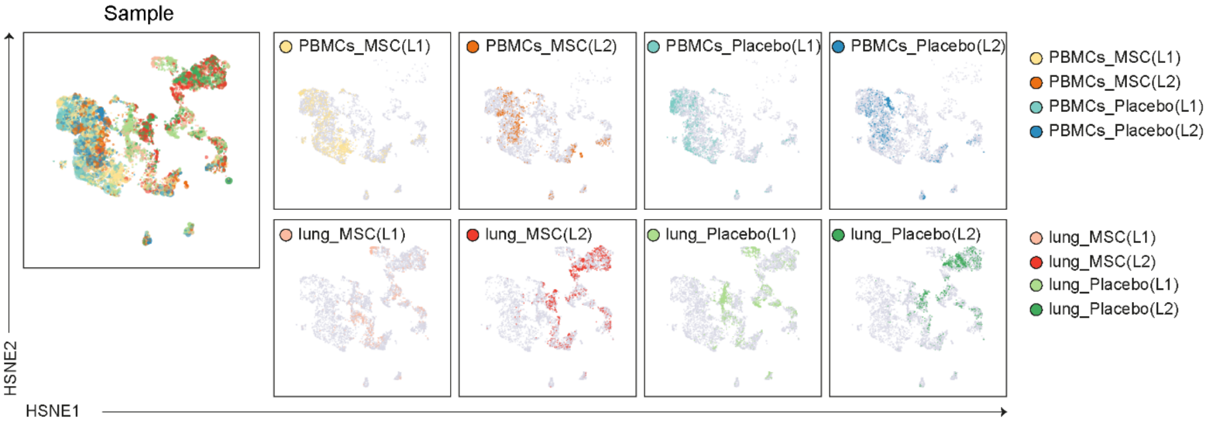

Figure S5. Cellular signatures reveal myeloid cell compartmentalization in PBMC and lung samples derived from L1 and L2.

A collective HSNE analysis was performed on PBMCs and lung-derived myeloid cell populations at L1 and L2, and identified cell clusters amongst the myeloid cells based on expression of myeloid-related markers. Colours represent samples derived from blood (PBMC - upper panel) or lung (lower panel) and L1 or L2 from emphysema patients who received BM-MSC/ placebo treatment.

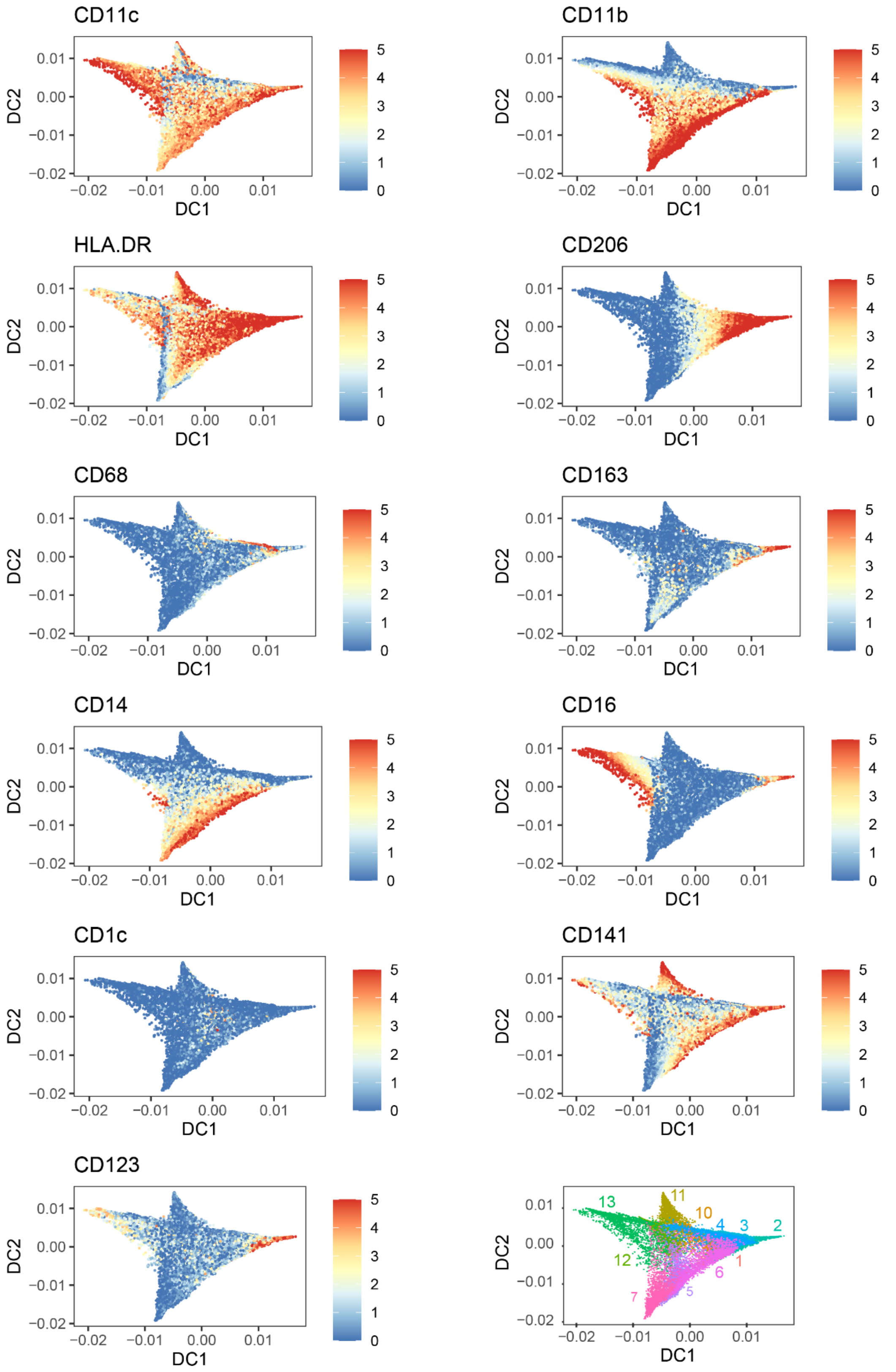
CD11c
CD11b
HLA.DR
CD206
CD68
CD163
CD14
CD16
CD1c
CD141
CD123
DC1
DC2
−0.02
−0.01
0.00
0.01
0
1
2
3
4
5
13
11
10
4
3
2
12
1
6
5
7

Figure S6. Expression profiles of individual myeloid markers were analysed using pseudotime analysis.

Pseudotime analysis of myeloid cells derived from PBMC and lung samples was conducted using Slingshot. The expression levels of individual myeloid-related markers are shown in the diffusion map. The colour-scale indicates the intensity of marker expression (red-high expression; blue-no/low expression). Each cluster within the myeloid cell population is distinguished by a unique colour and number.
